# Reactive Gas Cluster Ion Beams for Enhanced Drug Analysis by Secondary Ion Mass Spectrometry

**DOI:** 10.1021/acs.analchem.4c02144

**Published:** 2024-09-13

**Authors:** Matija Lagator, Bilal Patel, Sadia Sheraz, Nicholas Lockyer

**Affiliations:** † Department of Chemistry, Photon Science Institute, The University of Manchester, Oxford Road, Manchester M13 9PL, United Kingdom; ‡ Rosalind Franklin Institute, Building R113 Rutherford Appleton Laboratory, Harwell Campus, Didcot, Oxfordshire OX11 0QX, United Kingdom

## Abstract

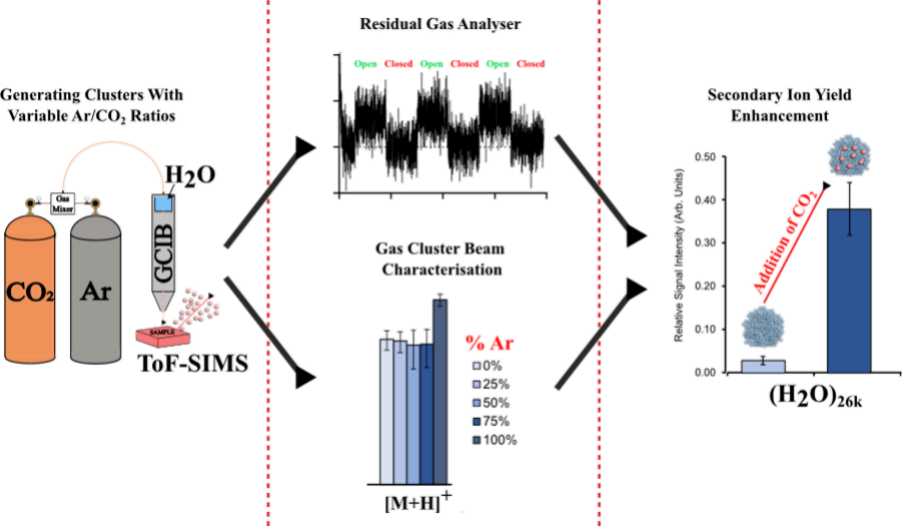

In this study, we investigate the formation and composition of Gas Cluster Ion Beams (GCIBs) and their application in Time-of-Flight Secondary Ion Mass Spectrometry (ToF-SIMS) analysis. We focus on altering the carrier gas composition, leading to the formation of (Ar/CO_2_)_n_ or (H_2_O)_n_ GCIBs. Our results demonstrate that the addition of a reactive species (CO_2_) to water GCIBs significantly enhances the secondary ion yield of small pharmaceutical compounds in the positive ion mode. In negative ion mode, the addition of CO_2_ resulted in either a positive enhancement or no effect, depending on the sample. However, an excess of CO_2_ in the carrier gas leads to the formation of carbon dioxide clusters, resulting in reduced yields compared to that of water cluster beams. Cluster size also plays a crucial role in overall yields. In a simple two-drug system, CO_2_-doped water clusters prove effective in mitigating matrix effects in positive ion mode compared to pure water cluster, while in negative ion mode, this effect is limited. These clusters are also applied to the analysis of drugs in a biological matrix, leading to more quantitative measurements as shown by a better fitting calibration curve. Overall, the doping of water clusters with small amounts of a reactive gas demonstrates promising benefits for higher sensitivity, higher resolution molecular analysis, and imaging using ToF-SIMS. The effectiveness of these reactive cluster beams varies depending on the experimental parameters and sample type.

Time-of-flight secondary ion mass spectrometry (ToF-SIMS) is a very powerful mass spectrometry imaging technique with applications including drug distribution studies.^1,2^ Of the various techniques for mass spectrometry imaging, ToF-SIMS offers the highest spatial resolution due to the ability to focus the primary ion beam to submicron dimensions. Additionally, a depth resolution of a few nanometers allows for high surface specificity and 3D imaging. Further advances in life sciences and other fields of application of the technique would be accelerated by addressing two major limitations for ToF-SIMS, low ionization yields and matrix effects. The former issue arises due to the low probability of a charge transfer or radical creation during primary ion impact. The ionization probability can vary greatly based on the analyte being sputtered and the primary ion beam, but usually it is in the range of 10^–3^ to 10^–5^ for organic secondary ions.^3−6^ This means that the vast majority of useful chemical information is lost due to the low likelihood of secondary ion formation. A similar situation arises in other mass spectrometry imaging techniques e.g. Matrix-Assisted Laser Desorption Ionization (MALDI).^7,8^ Improving secondary ion efficiency while limiting sample damage will facilitate the use of more highly focused primary beams and improved imaging performance. In SIMS there have been many different attempts to remedy this: matrix application,^9,10^ metal coating of samples,^11,12^ cryogenic condition and ice film formation,^13,14^ doping reactive compounds into the analysis chamber,^15,16^ laser postionization,^17,18^ etc. Although each of the methods has proved successful, they have associated limitations for 3D analysis or introduce additional technical or practical challenges, so further solutions are sought that can be widely applied to different samples.

Matrix effects represent a phenomenon of signal enhancement/suppression as a consequence of the chemical environment that is being analyzed and are common to most mass spectrometry imaging techniques.^19,20^ Namely, surrounding compounds will affect the likelihood that the compound of interest will be ionized. It has been hypothesized that this is due to competition for charge and that by providing more proton sources for charge transfer the matrix effects might be reduced in SIMS.^21,22^ It has been demonstrated that the gas-phase basicity of the analyte plays a role in this process.^22^ However, compared to low ionization yields, the issue of matrix effects is more difficult to adequately address or even measure in complex biological samples. This is due to the vast number and complex nature of compounds and the potential interactions between them. It is for this reason that most research on matrix effects is done with simple, often two-component, experimental systems.^18,23^ Various methods for ameliorating matrix effects in ToF-SIMS have been developed: change in primary ion (PI), particularly the introduction of water clusters;^15,21,22,24,25^ data correction methods;^26,27^ laser postionization,^28^ etc. However, none of these methods has been shown to resolve the problem completely by removing all potential matrix effects from complex biological samples. It has been necessary to develop new methods of dealing with this issue.

In order to increase molecular sensitivity and to reduce the degree of molecular damage to allow for depth profiling and 3D imaging of the sample by application of higher ion doses (beyond the static limit^29^) polyatomic PIs were adopted (C_60_,^30,31^ SF_6_,^32,33^ etc.). This trend was continued with the application and development of argon gas cluster ion beams (GCIB), with further reduction in surface damage and increases in molecular sputter yield, defined as the mean number of a molecular species removed intact from the surface per incident primary ion.^34^ The introduction of more reactive GCIB species (CO_2_^35^ and H_2_O^24^) proved to be particularly beneficial for ToF-SIMS analysis. It has been hypothesized that by increasing ionization yields, a secondary effect of reducing competition between analytes on the surface of interest results in lower matrix effects.^21^ This is because more of the sputtered species will be ionized, and so there will be less enhancement/suppression between the analytes. Supporting this hypothesis, water clusters have been shown to reduce matrix effects while at the same time increasing secondary ion yields.^15,21,22,24,25^ This is assumed to be achieved by providing a large supply of protons that can react with the released neutrals within the sputter crater or in the plume, thus promoting ionization.^36^ Molecular dynamics simulations suggest that protons from primary ion projectiles are most effective at promoting secondary ionization if they are more easily liberated than protons from the sample.^37^ This hypothesis would both explain the apparent reduced matrix effects and support the chemical modification of water GCIBs to facilitate proton formation i.e. acidification.

Doping of GCIBs has shown the potential to beneficially alter the beam characteristics. Incorporating small amounts of substances like water^21^ or methane^38^ into argon clusters has resulted in increased secondary ion yields. This is likely due to the higher reactivity of water and methane along with the presence of available protons or other charged species that are transferable to surface analytes. Similarly, introducing HCl to argon clusters while doping the surface of interest with water molecules from the gas phase also led to enhanced yields. This is theorized to occur due to the acidification of water, increasing the likelihood of proton donation.^15,39^ Although water clusters impacting kiloelectronvolt (keV) total energies will autoionize to some extent, facilitating easier proton transfer by chemical modification could further amplify ion yields and reduce matrix effects. Water cluster ion sources for SIMS often employ a carrier gas to facilitate large cluster formation by entraining water molecules in an adiabatic expansion through an orifice separating a high-pressure source region from a lower-pressure expansion region. Doping the carrier gas of water clusters is aimed to induce interactions between water and the carrier gas, resulting in more reactive clusters. Nilsson et al. examined doping water clusters with pure CO_2_ gas, yielding limited benefits for analysis of lipids in biological tissue.^40^ In contrast, Lagator et al. explored a similar approach but with a lower CO_2_ carrier gas concentration (12% in Ar), resulting in very significant increased yields for small drug molecules.^41^ While these differences could also be attributed to matrix effects, we propose that differing cluster formation processes due to different carrier gas compositions were responsible. To support primary ion beam developments leading to improved mass spectrometry imaging capability, a systematic study of carrier gas effects on GCIB composition and the resulting SIMS characteristics is required.

Results presented here involved the use of a residual gas analyzer (RGA) on the sample analysis chamber (SAC) to evaluate the composition of GCIB clusters. The purpose was to establish a link between the input gases used for cluster formation and the yields in secondary ion mass spectrometry, as well as to infer the mechanism of any secondary ionization enhancement. Previous studies have indicated that the ratio of Ar to CO_2_ gases plays a crucial role in cluster formation within PI mixtures.^42^ Lee et al. utilized an RGA to illustrate that input gas mixtures containing more than 10% CO_2_ predominantly led to the formation of (CO_2_)_n_ clusters. Our experiments support this finding, utilizing the RGA method for (Ar/CO_2_)_n_ clusters. We extended this methodology to water cluster primary ions by employing varying ratios of Ar and CO_2_ as carrier gases. The combined RGA/SIMS analysis in our study not only highlights the advantages of cluster doping but also sheds light on the underlying formation mechanism. The SIMS performance characteristics of GCIBs containing Ar, CO_2_ and/or H_2_O are then compared with regard to ion yield and matrix effects on drug-containing samples.

## Methods

### Sample Preparation - Drug Standards

Drug standards of acetaminophen (Sigma-Aldrich) and diclofenac sodium salt (Sigma-Aldrich) were dissolved in methanol (Honeywell, Riedel-de Haën) at a concentration of 10^–2^ M. Additionally, a 1:1 (v:v) mixture of the two compounds was prepared. Silicon wafers (5 × 5 mm^2^, Agar Scientific Limited) were placed in glass vials and cleaned using a sonicator (Elmasonic S 40, Elma). The sonication cleaning was done using consecutive washes in either methanol or water as well as a UV/Ozone cleaner (further details in Supporting Information (SI)). Solutions of the drug standards were deposited on the clean wafers by spin coating (further details are given in SI). Once all the solution had been added and dried, the silicon wafer was attached to a custom-made sample stub using conductive carbon tape. Diclofenac sodium salt samples were washed for 5 s in solution of ammonium formate (1.5 × 10^–3^ M, Sigma-Aldrich) and then allowed to air-dry again, prior to being attached to the sample stub. The samples were allowed to equilibrate for 24 h in the ToF-SIMS system to reduce the influence of signal loss due to sample removal by the vacuum.

### Sample Preparation - Tissue Sections

A sample of commercially available porcine liver as well as mouse brain tissue was sectioned using a cryostat (Leica CM3050 S Cryostat). The mice were sacrificed at the Wolfson Molecular Imaging Centre (WMIC) in accordance with ethical guidelines described by the Animals (Scientific Procedures) Act 1986 and the UK Home Office. The tissue was mounted by using 15% carboxymethyl cellulose. This was done so that only one side was embedded and attached to the sample holder, leaving the other side frozen and not touching the embedding matrix. The thickness of the sectioned slices was 10–12 μm, and the cryotome was at −20 °C during the procedure. The liver slices were thaw-mounted on clean silicon wafers (5 × 5 mm^2^, Agar Scientific Limited) while brain tissue was mounted on cut indium tin oxide (ITO) coated glass slides. All tissue was stored at −80 °C. Stock solution (100 mM) of acetaminophen (Sigma-Aldrich) in methanol was prepared. This stock solution was used to prepare a dilution series of concentrations: 10, 5, 2.5, 1.25, and 0.625 mM. Prior to the mass spectrometry experiments, the sectioned tissues were removed from the −80 °C freezer and desiccated for 15 min. For liver tissue, a micropipette was used to add 3 μL of each of the solutions to a separate tissue section. The sections were desiccated again for 15 min in order to allow for the methanol to evaporate and only for the drug to stay on the tissue. For the brains sections 2 μL of each solution were added to a single brain hemisphere. The tissue was allowed to airdry. The silicon wafers and ITO slides with the tissue sections were attached to a sample stub by using conductive carbon tape and transferred into the ToF-SIMS instrument.

### Mass Spectrometry Analysis

After a period of 24 h in vacuum the samples were analyzed using the ToF-SIMS (J105 3D-Chemical Image, Ionoptika Ltd.) described previously.^43^ GCIBs were formed using custom mixes of Ar/CO_2_ directed through a water cluster source, which when operated provided the possibility of forming water-containing GCIBs with Ar/CO_2_ acting as a carrier gas.^24^ Custom Ar/CO_2_ mixes were restricted in delivery pressure to <8 bar, limiting the available cluster size (*n* < 3000). Separately, a premixed gas cylinder (86% Ar, 12% CO_2_, 2% O_2_) was used up to 18 bar to form larger GCIBs. GCIBs (70 keV) were mass-selected using a Wien filter in the ion column. For comparison, data were also collected with a separate 40 keV C_60_ primary ion beam (Ionoptika Ltd.) on the same instrument. Parameters that were used for analysis can be seen in Tables S1–S7. For each experiment, the ion dose per layer was adjusted to comparable values for each primary ion. Putative assignments of measured peaks are given in the Supporting Information (Table S8).

Note that there are four separate sets of ToF-SIMS experiments:

1) Table S1, S2, S3, and S4 - Standards analyzed in positive ion mode using gas mixer (varioMix, Ibeda) to explore multiple GCIB composition with limited cluster size.
2) Table S5 - Standards analyzed in both positive and negative ion mode using premixed gases ordered from BOC. These served as carrier gases for larger water clusters. The gases used were Argoshield Universal (86% Ar, 12% CO_2_, 2% O_2_) and Pureshield Argon (99.998% Ar).
3) Table S6 - Pork liver tissue sections analyzed in positive ion mode using GCIBs formed from Argoshield Universal (BOC) and carbon dioxide (99.80% CO_2_) gases, as well as using a C_60_ primary ion.
4) Table S7 - Mouse brain tissue sections analyzed in positive ion mode using water clusters with the Ar/CO_2_ carrier gas (86% Ar, 12% CO_2_, 2% O_2_)

### Residual Gas Analysis (RGA) Experiments

A residual gas analyzer (HPR-30 Vacuum Process Gas Analyzer, Hiden Analytical) was used to analyze the gas that was present in the SAC of the ToF-SIMS instrument and to determine any changes due to the operation of the GCIB. Measurements of *m*/*z* 18 (H_2_O), 40 (Ar) and 44 (CO_2_) were taken using the RGA. The gas cluster ion beam on the ToF-SIMS was used with 90% duty cycle (close to DC mode) to sputter a gold sample. For background RGA measurements the GCIB was physically blanked by closing the in-column gate valve, and the RGA data gathering would proceed in continuous mode. After 2 min the gate valve was opened, and analysis continued for an additional 2 min after which the gate valve was shut again. This cycling between the opened and closed gate valve was repeated three times. The averages of the opened and closed periods of data gathering were calculated. A period of 10 s before and after the 2 min mark were ignored to allow time for gas concentration to reach a steady state.

### Data Processing

All the SIMS data were analyzed using Analyzer Software (Ionoptika Ltd.). For each experiment, the peaks of interest were selected with a consistent width of the selection tool. While all the SIMS data was collected using 32 × 32 pixels it was necessary to select smaller areas within these analyzed regions. This is a consequence of the ultrahigh vacuum in the ToF-SIMS instrument that removes drug sample over time, thus leaving the sample surface patchy. In order to have a fair representation of the intensity several regions with highest intensity were selected on each analyzed area (further details in SI). The chosen peak was integrated to arrive at the area under the curve, which was representative of signal intensity. This was repeated over the number of data acquisition layers that were collected. When analyzing drug standards, the data from the top 10 layers were disregarded in order to reduce the influence of surface contamination as well as to allow for the signal to reach a position that is closer to the steady state of signal intensity. For tissue samples all 30 layers were analyzed as signal levels were low. The intensities of the selected layers were summed and used for further calculations, which were done using Microsoft Excel. For the plots showing the relative yields, the total integrated signal intensity was divided by the total primary ion dose to arrive at “Relative Signal Intensity (Arb. Units)”. For matrix effect analysis, the yields of acetaminophen (Ace) were divided by the yields of diclofenac sodium salt (Diclo). This was done separately for pure and mixed drug standards (both drugs on the same wafer). These Ace/Diclo ratios were divided by each other: Pure Ace/Diclo ratio divided by Mixture Ace/Diclo ratio. This value presented the discrepancy between the pure and mixture relative yields and was representative of matrix effects. Relative signal intensities were statistically compared by using an unpaired *t* test in the GraphPad *t* test calculator software (https://www.graphpad.com/). Error bars shown in the figures are representative of a 95% confidence interval.

## Results

### Characterization of GCIB Projectiles

To indicate the composition of GCIBs with varying Ar and CO_2_ input gas ratios, the change in argon (*m*/*z* 40) concentration in the analysis chamber between the “closed” and “opened” states of the GCIB gate valve was measured with the RGA. The background levels of argon should remain consistent for all gas compositions while the valve is closed. However, over time, the baseline argon level shifted, likely due to slight pressure variations within the instrument and residual argon from previous gate valve openings (Figure S2). As anticipated, using pure argon as the input gas (100% Ar) for the GCIB resulted in a pronounced increase in the *m*/*z* 40 RGA signal when the GCIB gate valve was opened, indicating that the clusters generated were primarily composed of argon (Figure S2). According to Lee et al., the Ar percentage in clusters decreased rapidly as the percentage of Ar in the ion source feed mixture decreased. This trend was such that beyond 10% CO_2_ in the input mixture, the resulting clusters consisted of over 95% CO_2_.^42^ However, our findings demonstrated that even at a 25% CO_2_ input ratio, observable amounts of Ar were still present in the clusters as indicated by the increased *m*/*z* 40 signal intensity when the valve was open. Decreasing the Ar percentage below 75% effectively resulted in no detectable Ar in the chamber (Figures S1 and S2). This is supported by the fact that in experiments with higher CO_2_ input content, the signal measurements were consistently higher in the “closed” position compared to the “open” position, implying that the detected argon signal was present as background gas rather than being the result of the GCIB gate valve opening. Additionally, in all measurements except one, argon levels were low enough to generate negative values, suggesting that the argon concentration was not sufficiently high for the RGA detection range. While the overall results are in agreement with those shown by Lee et al., differences in ion source designs and gas dynamics could potentially explain the discrepancy in the cluster composition and relative stability.

The application of various Ar/CO_2_ ratios in carrier gases to water clusters yielded less conclusive outcomes for the RGA analysis (Figures S3 and S4). There was minimal variation in the relative signal intensities of *m*/*z* 40, and unlike the clear increase observed when Ar/CO_2_ was used as the PI, no distinct trend emerged. This could be attributed to the higher presence of water in the clusters, reducing the likelihood of argon atoms. Attempts to detect water molecules (*m*/*z* 18) using RGA did not yield significant differences between open and closed gas valve states (Figure S3). This is likely due to the background water levels in the chamber being too high for the clusters to cause an observable difference. Water signal did climb over time for all of the carrier gas mixtures, and this was at an observably higher level for the 100% Ar experiments (Figure S3). A potential implication of these findings is that while there is no clear increase with opening of the gas valve, there is a possibility of water buildup in the analysis chamber over time while operating the water source, which could also be beneficial for ToF-SIMS analysis. This requires further investigation, for example, performing SIMS analysis with the C_60_ beam while having the water source at operating temperature but electronically blanked. In summary, RGA analysis of gases during water cluster utilization was inconclusive and would require more sensitive methodology for further investigation. Potentially this could be addressed by repositioning the RGA closer to the outlet of the GCIB column or reducing the base pressure and/or the pumping speed in the SAC.

**Figure 1:**
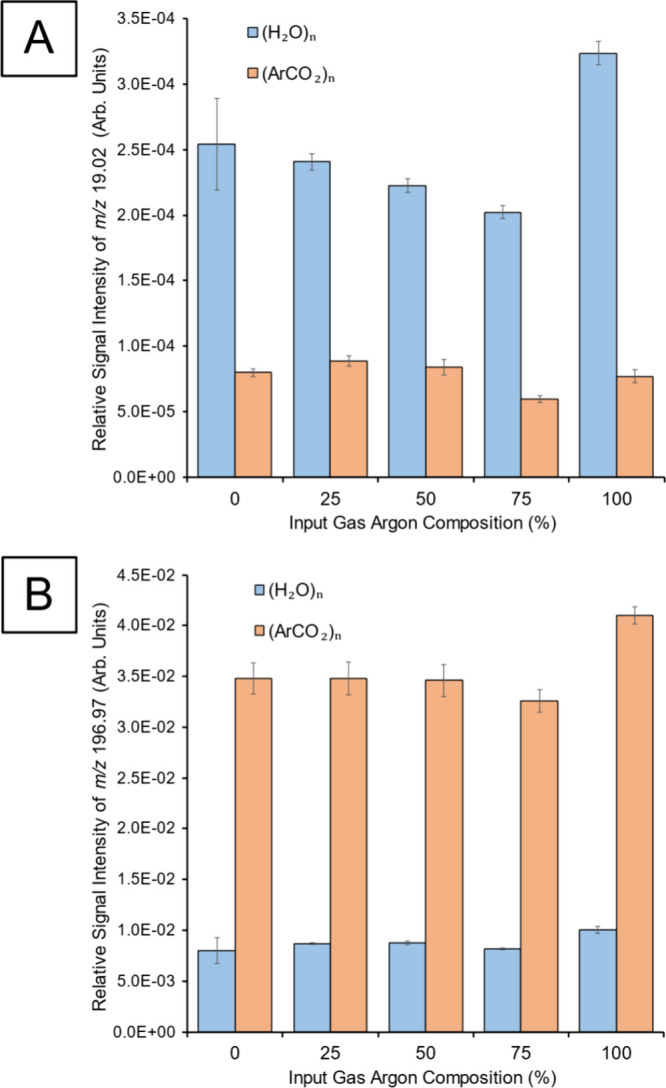
Showing effects of different ratios of Ar and CO_2_ when used as either primary ions (“(ArCO_2_)_n_”) or carrier gases for small water clusters (“(H_2_O)_n_”). All analyses were done on a gold surface. (A) SIMS results for *m*/*z* 19.02 [H_3_O]^+^ and (B) for *m*/*z* 196.97 [Au]^+^. Cluster *E/m* ∼ 0.8 eV/nucleon for (ArCO_2_)_n_ and ∼1.6 eV/nucleon for (H_2_O)_n_ (n ∼ 2100 and 2400 respectively for (Ar/CO_2_)_n_ and (H_2_O)_n_).

While the RGA was not able to pick up any differences in water cluster gas composition (Figure S4), the ToF-SIMS results from gold samples provide an indication of the cluster composition over a range of different carrier gases (Figure 1). The *m*/*z* 19 [H_3_O]^+^ water-related peak showed no significant change in signal intensity when changing the Ar/CO_2_ gas feed composition without filling the water reservoir in the GCIB source (“(ArCO_2_)_n_” data) (Figure 1A). This was a good indication of a negative control, since there should not have been any change in water signal levels in the sample since no water was used in cluster formation. Using the dry (ArCO_2_)_n_ PIs the Au^+^ signals are consistent for all CO_2_-containing gases with a small increase when using Ar_n_ GCIBs (Figure 1B). With the water source operating, the [H_3_O]^+^ signal was increased compared to that of (Ar/CO_2_)_n_ PIs. The [H_3_O]^+^ signal intensity was within experimental error for all of the GCIB carrier gas compositions except for 100% Ar, where an increase was observed. A comparable result is observed for the Au^+^ signal. Proton transfer will not directly affect the efficiency of Au^+^ formation, and the reduced yield of this species when operating the water source can be explained by a reduced atomic sputter yield for (H_2_O)_n_ GCIBs compared to (Ar/CO_2_)_n_. This is consistent with the nature of the surface analyzed, being hard, inert, and yielding metallic ions. Lee et al.^42^ report the sputter yield of Si at ∼0.2 eV/nucleon is reduced by >80% when switching GCIB feed gas from Ar to >38% CO_2_, a result which the authors attribute to a reduction in the atomic masses of constituent atoms in the clusters. The results in Figure 1B show the same trend for Ar_n_ and (Ar/CO_2_)_n_ albeit with a smaller difference, which could be due to our increased eV/nucleon. A further reduction in signal (or sputter yield) when operating the water source could be explained by molecular mass difference between H_2_O and CO_2_ as concluded by Sano et al.^44^

The results in Figure 1 are consistent with a mixed H_2_O/CO_2_ cluster when the carrier gas is 0–75% Ar and the water source is operated. With a carrier of 100% Ar the GCIB composition is different but also contains H_2_O. When the water source is not operated the Ar/CO_2_ feed gases produce GCIBs with a fairly uniform composition with 25% or greater CO_2_ in feed, which behave differently to the Ar_n_ GCIBs formed from the pure Ar feed. This also supports the RGA results with Ar/CO_2_ as the primary ions (Figure S2). Although a detailed theoretical consideration of GCIB formation is beyond the scope of this study, it is instructive to consider the relevant Condensation parameters (*K*) which in turn feature in Hagena’s empirical scaling laws for cluster formation.^45^ These values are 1650,^46^ 3660^47^ for Ar and CO_2_ respectively. Higher *K*-values imply more favorable cluster formation. Source pressure and temperature as well as nozzle design are other important parameters.^48^ Previously Moritani et al.^49^ reported that mixed Ar/CH_4_ feed gases produced GCIBs which were predominantly (CH_4_)_n_ when the %CH_4_ was 10% or higher. Wucher et al.^38^ also explained a reduction in secondary ion yield from Ar/CH_4_ mixtures with >3% CH_4_ as the result of a change in GCIB composition. We note that the condensation parameter (*K*) for CH_4_ is 2360.^46^ The shift from Ar_n_ toward more stable (CH_4_ or CO_2_) GCIBs observed in our work and that of others is therefore supported by theory. An alternative explanation for the increase in [H_3_O]^+^ signal when the water source is operating is that water is depositing on the gold sample from the gas phase and the primary ions contain only Ar and/or CO_2_. The results in the following section provide further evidence that H_2_O is incorporated into GCIBs by using feed gases containing CO_2_.

An additional observation from the gas mixing experiments with water clusters is that changing the Ar/CO_2_ carrier gas ratio affected the spot size. While this change was relatively small (Figure S5) there is a clear pattern that with a greater percentage of CO_2_ in the carrier gas, the lateral resolution improves (smaller spot size). The improved lateral resolution of (CO_2_)_n_ and (Ar/CO_2_)_n_ GCIBs compared to Ar_n_ has been reported previously^35,42^ and has been linked with their higher binding energy and greater stability.^35^

### SIMS Characterization of Drug Standards

**Figure 2:**
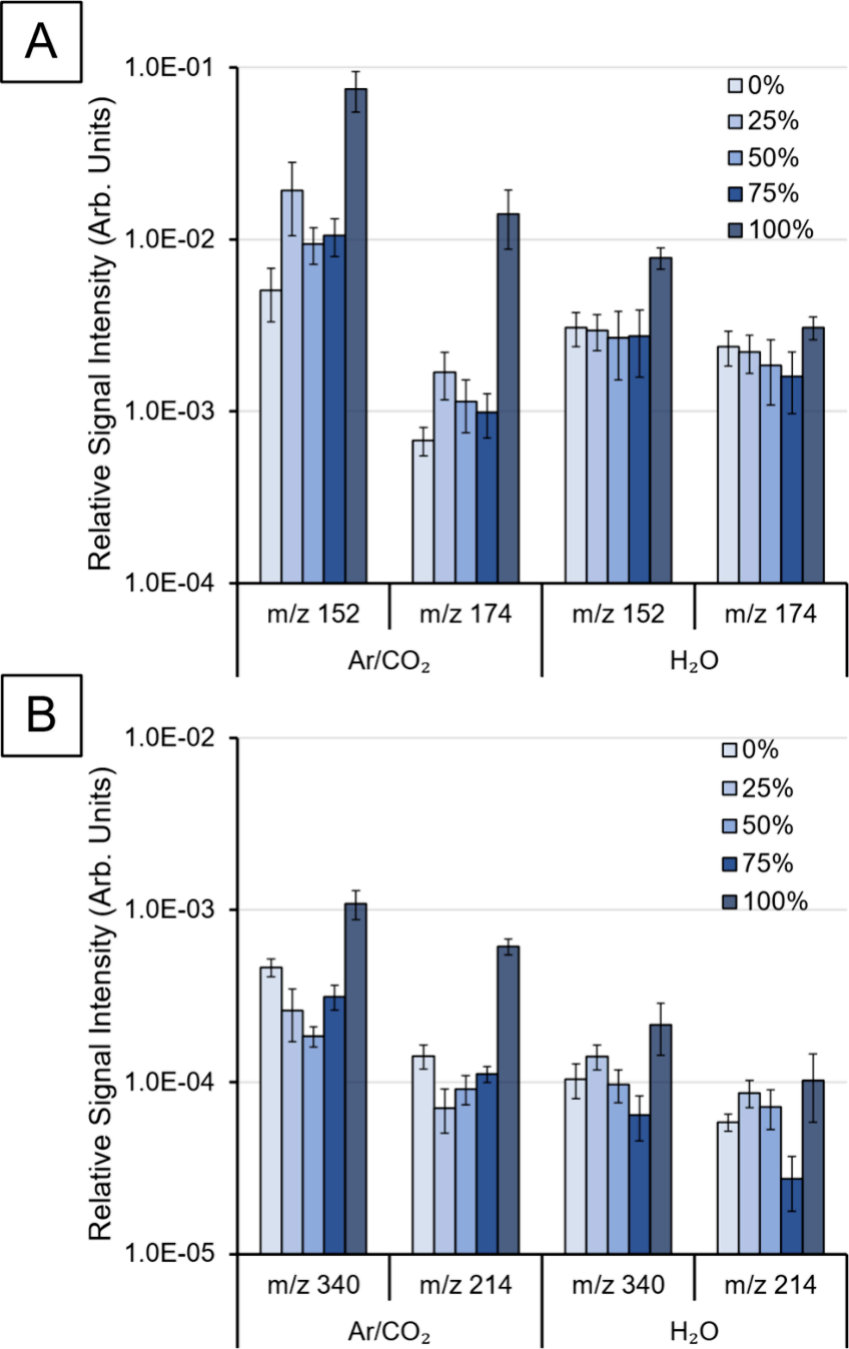
Showing the relative signal intensity (normalized to total primary ion dose) of (A) acetaminophen *m*/*z* 152 [M + H]^+^, 174 [M + Na]^+^ and (B) diclofenac sodium salt, *m*/*z* 340 [M + Na]^+^, 214 [M-CO_2_-Cl]^+^ as a function of different ratios of Ar and CO_2_ input (“Ar/CO_2_”) and carrier gases for water clusters (“H_2_O”). The percentages represent the amount of argon in the gas mixture. Cluster *E/m* ∼ 0.9 eV/nucleon for each primary ion beam (n ∼ 2000 and 4200 respectively for (Ar/CO_2_)_n_ and (H_2_O)_n_).

The same procedure that was used to analyze the gold sample (Figure 1) was applied to pure standards of acetaminophen and diclofenac sodium salt on silicon wafers (Figure 2). These compounds were chosen because they are small, relatively simple, and frequently used pharmaceuticals, forming different types of secondary ion. For Ar/CO_2_, with no water, the pattern of the drug signal intensities is similar to the pattern observed for gold ions in Figure 1B; i.e., the signal intensity for CO_2_ containing gases is quite constant considering experimental error but is significantly greater for 100% Ar input gas composition. This is true for all of the four analyzed peaks (acetaminophen – *m*/*z* 152 [M + H]^+^, 174 [M + Na]^+^; diclofenac sodium salt – *m*/*z* 340 [M + Na]^+^, 214 [M-CO_2_Na-HCl]^+^). A possible explanation for this is that with Si targets for pure argon clusters the sputter yields are greater than that for carbon dioxide clusters for these small molecules, as shown by Lee et al.^42^ and discussed above. When the water source is operational with the different carrier gases, we observe the following. Once again, the CO_2_-containing gases (0–75% Ar) yield similar results for each of the measured signals. The 100% Ar carrier gives a significant signal increase for *m*/*z* 152 [M + H]^+^ compared with the CO_2_ mixtures. Compared with the dry clusters without operating the water source, the like-for-like signal levels are reduced for 100% Ar while the CO_2_ mixtures show no clear trend. These results support the previous conclusion that 0–75% Ar gases form clusters with similar characteristics and compositions, which are different from clusters formed from 100% Ar. The incorporation of H_2_O into clusters is implied by consideration of signal levels with/without operation of the water source. Signal levels will be dependent on both GCIB sputter yields and secondary ionization efficiency.

Due to the limited pressure range of our gas-mixing apparatus these gas composition studies were again restricted to smaller clusters, above the *E/m* range where the greatest ionization enhancement has been observed for (H_2_O)_n_.^50^ We note also that Sano et al. report sputter yields using (H_2_O)_n_ < (CO_2_)_n_ ∼ Ar_n_ for the larger Irganox 1010 molecule.^44^ Taken together, this would explain why secondary ion yields appear to be lower for (H_2_O)_n_ than for Ar_n_ (100% Ar beams). Sputter yield comparisons for small molecules using (H_2_O)_n_ and (CO_2_)_n_ are not readily available but if behavior mimics that observed on Si samples by Lee et al.^42^ that would explain why (CO_2_)_n_ clusters give lower signals than Ar_n_. Further measurements of sputter yields and secondary ion yields for different GCIBs on a range of molecular samples are needed to further our understanding of the effects of mass in the projectile and analyte. To recap, we have demonstrated that gas mixes containing somewhere between 0% and 25% CO_2_ in Ar form predominantly (CO_2_)_n_ clusters and that these gases form mixed (CO_2_/H_2_O)_n_ clusters when used as carrier gases for the water GCIB source.

**Figure 3:**
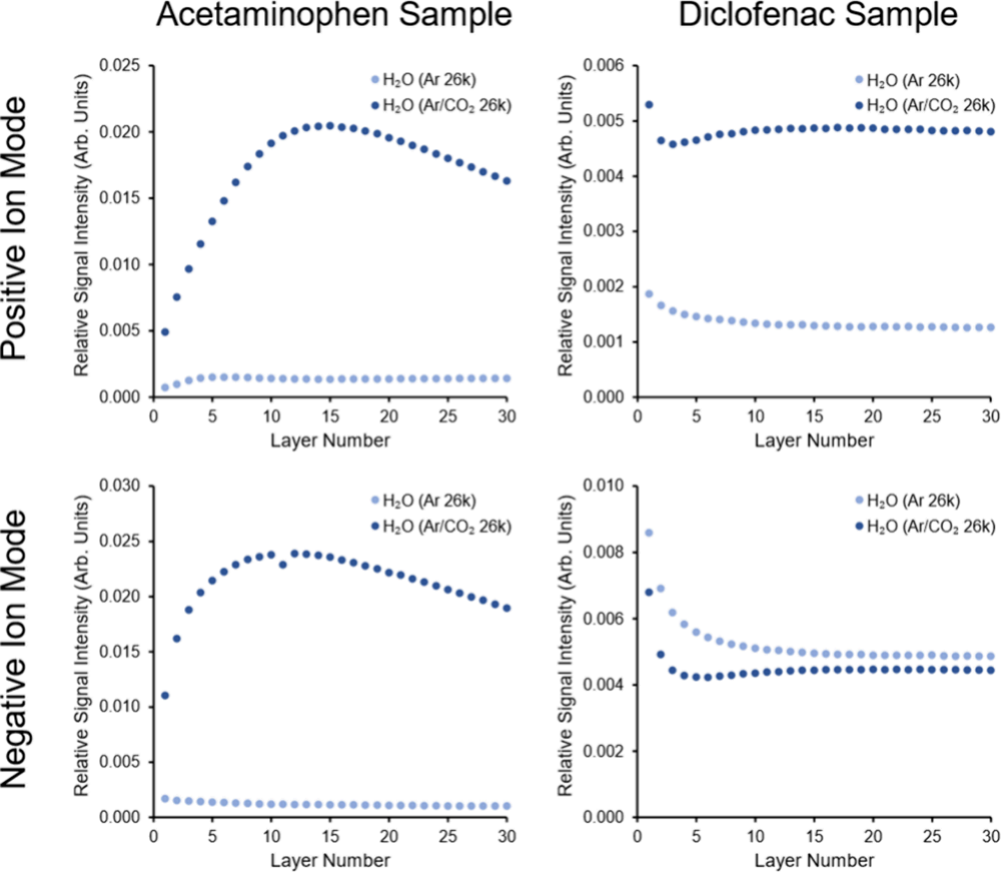
ToF-SIMS depth profiles of acetaminophen and diclofenac thin films acquired with two (H_2_O)_n_ PIs with different carrier gases. Experiments were repeated in positive and negative ion mode. Total primary ion dose for the 30 layers is ≈1.2 × 10^13^ ions/cm^2^. Cluster *E/m* ∼ 0.15 eV/nucleon for each primary ion beam (n ∼ 26000 for both (H_2_O)_n_ with Ar and ArCO_2_ carrier gas). In both positive and negative ion modes a sum of all analyzed drug-specific peaks is plotted. These are Acetaminophen/Positive – *m*/*z* 152, 109, 110, 174, 196, 303, and 325; Acetaminophen/Negative – *m*/*z* 150, 107, and 301; Diclofenac/Positive – *m*/*z* 340, 342, 318, 320, 322, 324, 296, 298, 214, and 216; Diclofenac/Negative – *m*/*z* 316, 318, 294, 296, 250, 252, 214, and 216.

**Figure 4:**
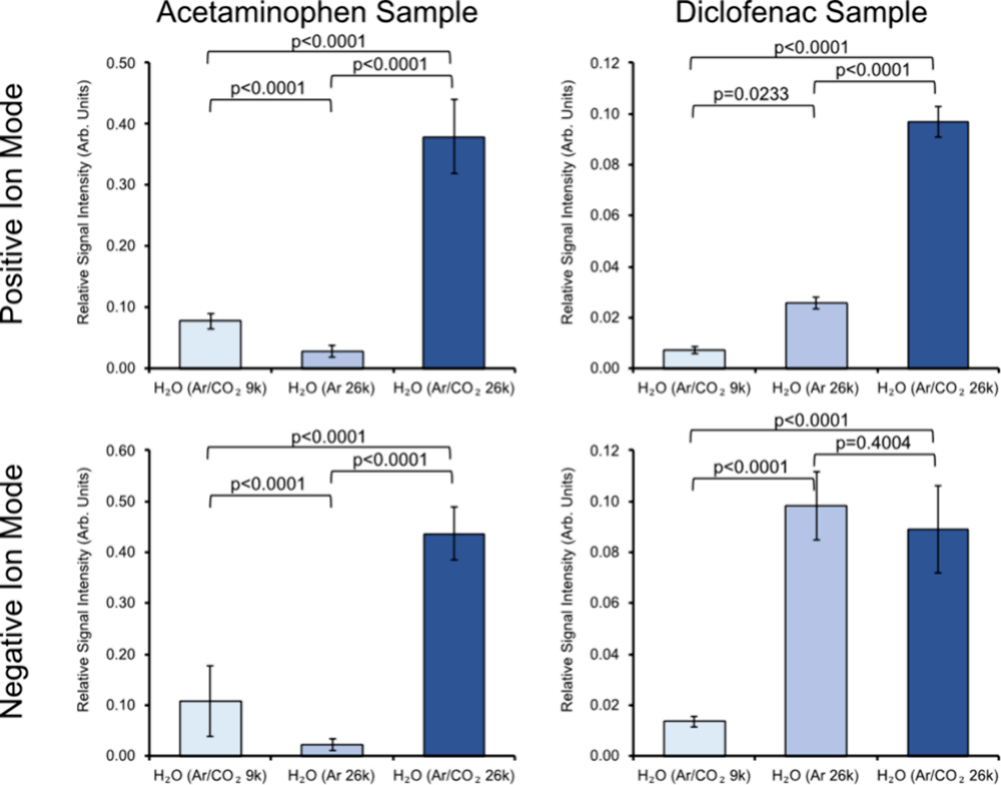
Comparing the relative ToF-SIMS ion yields of acetaminophen and diclofenac using pure Ar as well as a mixture of Ar (86%) and CO_2_ (12%) as carrier gases for water cluster PI. Cluster *E/m* ∼ 0.15 eV/nucleon for larger primary ion beams (n ∼ 26000 for both (H_2_O)_n_ with Ar and ArCO_2_ carrier gas) and *E/m* ∼ 0.41 eV/nucleon (n ∼ 9400) for smaller clusters. Experiments repeated in positive and negative ion mode. Yields are sums of all observed fragments and molecular ions for layers 11–30 of a depth profile acquired with a cumulative primary ion dose of ≈0.8 × 10^13^ ions/cm^2^. These are Acetaminophen/Positive – *m*/*z* 152, 109, 110, 174, 196, 303, and 325; Acetaminophen/Negative – *m*/*z* 150, 107, and 301; Diclofenac/Positive – *m*/*z* 340, 342, 318, 320, 322, 324, 296, 298, 214, and 216; Diclofenac/Negative – *m*/*z* 316, 318, 294, 296, 250, 252, 214, and 216.

Our lab has previously established that water clusters are particularly effective at increasing SIMS yields at large cluster size (*E/m* less than ∼0.2 eV/nucleon).^50^ Results of SIMS analysis using a premixed cylinder of gases (86% Ar, 12% CO_2_, 2% O_2_) were compared to ToF-SIMS yields obtained from pure Ar input gas both of which were used as carrier gas for water clusters (Figures 3 and 4). This allowed for larger GCIB clusters to be studied, as the delivery pressure could be increased without needing the gas mixer. For all experiments 30 layers (total ion dose ≈1.2 × 10^13^ ions/cm^2^) were collected (Figure 3) to account for potential surface contaminations which are a frequent issue in ToF-SIMS analysis and to determine any changes to secondary ion yield as a function of primary ion dose. The sum of all analyzed peaks for each of the drug standards was used to produce the depth profiles shown in Figure 3 (see figure legend for complete list). Based on the depth profiles (Figure 3) it was decided that a sum of layers 11–30 would provide a more representative indication of the overall efficiency of the PIs. This minimizes any sample contamination effects and also reflects the analytical situation in which GCIBs are mostly applied, at relatively high dose for subsurface measurements. The condensation parameters (*K*) for O_2_ is 1400^47^ (less that that of Ar) so we consider the contribution of O_2_ to the cluster formed from the premixed gas to be negligible.

The results shown in Figure 4 clearly demonstrate that the addition of CO_2_ to the carrier gas greatly influences the relative signal intensities of these analytes. An overall increase ×2.3–31 in ion yield was observed with addition of CO_2_ for both drugs in positive ion mode and for acetaminophen also in negative ion mode. For diclofenac, a potential explanation for the lack of a significant difference in negative ion mode between large water clusters with different carrier gases could be the fact that diclofenac salt is expected to pre-form ions on the Si surface. Considering secondary ions formed via proton transfer processes, by doping the water (p*K*_a_ ∼ 14) clusters with CO_2_, it can be expected that carbonic acid (H_2_CO_3_, p*K*_a_ ∼ 6 in water) is formed, thus enhancing the cluster’s ability to act as a source of [H]^+^ ions, or a sink for [H]^+^ ions in reaction with conjugate base ions ([HCO_3_]^–^). In positive ion mode, while some protonated diclofenac molecular ions were observed, the majority of detected molecular ions were sodiated. The solvation of sputtered secondary ions using water GCIBs may be an important stabilization mechanism, preventing reneutralization and promoting Si yields. It is unclear why CO_2_ doping appears to enhance sodiated ions more that pure water clusters, for both analytes (Figure S9). Further study is required to corroborate this observation.

As observed previously with water GCIBs^21,51^ changing the *E/m* of the cluster can greatly affect the overall yield. The optimum *E/m* value depends on both the cluster type and the sample that is being analyzed. When comparing larger (n ∼ 26,000, *E/m* ∼ 0.15 eV/nucleon) and smaller (n ∼ 9000, *E/m* ∼ 0.41 eV/nucleon) water clusters with the Ar/CO_2_ carrier gas, the larger clusters result in higher yield in each case (Figure 4). Our data are consistent with previous studies showing that for small molecules (<400 Da) the secondary ion yield with water clusters is maximized at *E/m* ∼ 0.15 eV/nucleon.^50^

In order to determine the capabilities of GCIBs to detect pharmaceuticals in biological matrices, a proof-of-principle study was carried out. Acetaminophen (3 μL) was doped at a range of concentrations up to 10 mM onto the surface of cryosectioned pork liver. Each concentration was doped onto a separate sample. These doped sections were analyzed using 5 separate PIs (Figure S6), and a calibration plot was constructed (Figure S7). From these data it could be observed that for acetaminophen and phosphocholine headgroup, the CO_2_ (pure) and H_2_O(CO_2_) GCIBs had very similar yields, consistent with the hypothesis that CO_2_ is the major constituent in both clusters which are quite different from clusters formed from Ar/CO_2_ (86% Ar, 12% CO_2_, 2% O_2_) mixtures. Water with pure CO_2_ carrier gas resulted in significantly lower yields for both measured secondary ions compared to the water with Ar/CO_2_ carrier gas (and also C_60_).

### Determination of Extent of Matrix Effects

**Figure 5:**
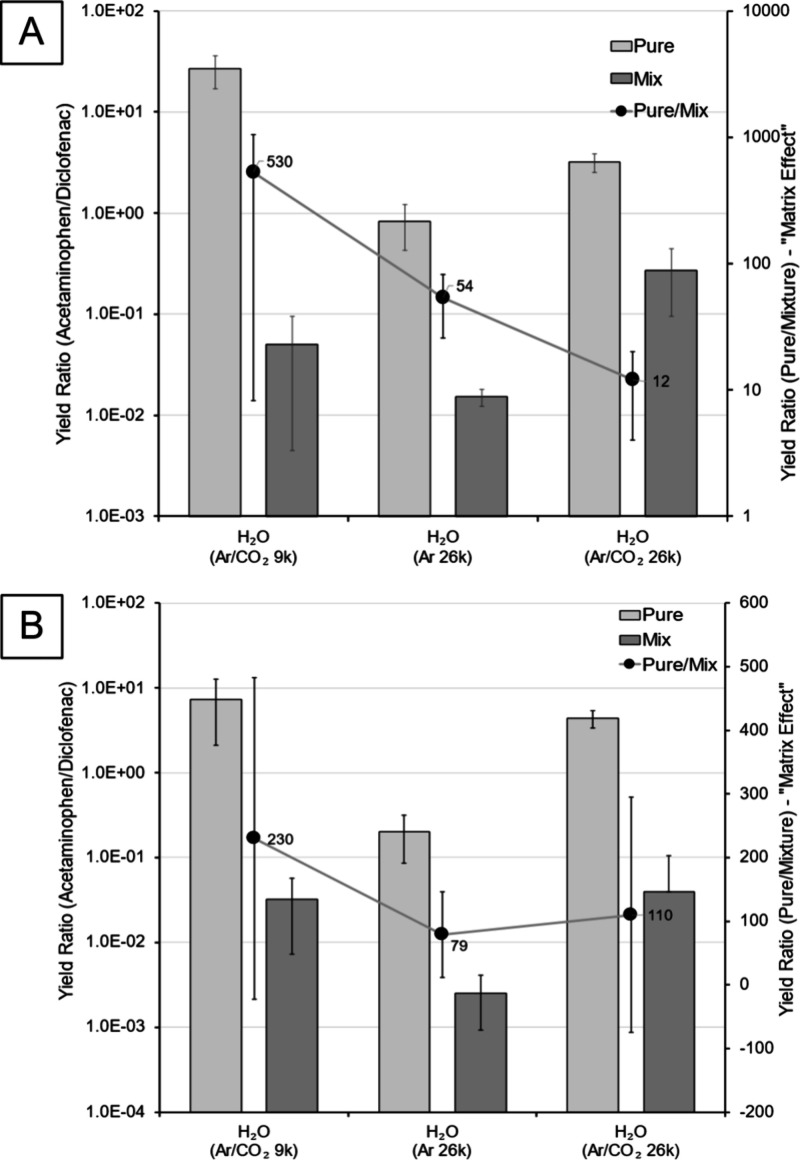
Matrix effects in positive (A) and negative (B) ion mode. Comparing the yield ratio of acetaminophen to diclofenac in both pure and mixture samples (primary *y*-axis). This is done for different primary ions, as shown on the *x*-axis. Cluster *E/m* ∼ 0.15 eV/nucleon for larger primary ion beams (n ∼ 26000 for both (H_2_O)_n_ with Ar and ArCO_2_ carrier gas) and *E/m* ∼ 0.41 eV/nucleon (n ∼ 9400) for smaller clusters. The ratio of pure to mixture yields is indicative of the severity of the matrix effects. This is represented by the black line (secondary *y*-axis). The yields are obtained by summing the signal intensity for all of the diagnostic peaks that were observed with all PIs. For positive ion mode, these peaks are Acetaminophen (*m*/*z*) – 152, 174, and 196; Diclofenac (*m*/*z*) – 340, 342, 318, 320, 322, 324, 296, and 298; for negative ion mode the peaks are Acetaminophen (*m*/*z*) – 150 and 107; Diclofenac (*m*/*z*) – 316, 318, 294, 296, 250, 252, 214, and 216.

Previously it has been shown that water clusters can lower the extent of matrix effects.^15,21,22,24,25^ In this study, we build on previous research by investigating the influence of cluster size and carrier gas on matrix effects observed with water GCIBs. This was done by comparing the yields of acetaminophen and diclofenac in both pure samples and 1:1 molar mixture (Figure 5). Due to inherent differences in ionization efficiency of various compounds, even in pure drug samples there was a substantial difference in signal intensities (Figure 4). Due to this natural difference in secondary ion yields of the compounds it was necessary to compare the signal intensity ratios from both pure drugs and drug mixtures in order to get a measure of the matrix effects. The more consistent the ion ratios of pure and mixture samples were, the less the interaction of the two compounds affected the respective ionization efficiencies, thus implying lower matrix effects. This was represented by the secondary *Y*-axis and the black line in Figure 5. In positive ion mode (Figure 5A), for water clusters, the data show a decrease in matrix effects by two factors:

1 Increasing the cluster size—both of the n ∼ 26,000 clusters have lower matrix effects than the smaller n ∼ 9000 clusters.
2 Changing the carrier gas for water clusters from pure Ar to Ar/CO_2_—for clusters of same size (n ∼ 26,000) adding CO_2_ to the carrier gas further decreased the matrix effects.

These two parameters appear to decrease matrix effects and to make the large water clusters with Ar/CO_2_ carrier gas the “most quantitative” GCIB for analysis of acetaminophen and diclofenac in the positive ion mode. In negative ion mode (Figure 5B) the situation is similar in that the smaller water clusters with Ar/CO_2_ carrier gas exhibit the greatest matrix effects but for the larger clusters the presence of CO_2_ increased the matrix effect compared to the pure water cluster of the same size. Overall, the variation (maximum matrix effect/minimum matrix effect values observed) in matrix effects (yield ratios) is much greater (∼×44) in positive ion mode than negative ion mode (∼×3). For example, the positive ion yield ratio with ArCO_2_ carrier gas is reduced from 530 (*n* = 9000) to 12 (*n* = 26000) (Figure 5A). This could potentially be explained by the increased ability of the reactive/acidic water clusters to donate protons due to the mixing of water and carbon dioxide. This would impact the results in the positive ion mode to a greater extent and could explain the discrepancy between the two ion modes. However, what is still clear is that increasing the water cluster size from 9000 to 26000 reduced the matrix effects in both ion modes. Note that there is a practical limit to how low these *E/m* values can go as at some point energy would be insufficient for sputtering, dependent on analyte mass.^44^

The ability of the water clusters to reduce matrix effects was also tested by doping biological tissue (a complex chemical environment). This is reflected in acetaminophen (*m*/*z* 152) calibration curves shown in Figure S7. From these data, it was observed that the large water clusters with Ar/CO_2_ carrier gas result in a calibration curve fit with the *R*^2^ value of 0.8014 when using a linear fit. Note also that the results of the water cluster with CO_2_ carrier gas are quite similar to those for CO_2_ as the PI. With both of these PIs the majority of the values are either close to 0 or even negative and the only increase is at 10 mM. These results further support the findings that using pure CO_2_ carrier gas results in clusters being formed that are mostly made of CO_2_. Additionally, note that for the H_2_O(Ar/CO_2_) clusters the yields are 3 orders of magnitude higher than that for the H_2_O(CO_2_) or the CO_2_ clusters. Lastly, results from doping mouse brain tissue with varying concentrations of acetaminophen resulted in a good linear fit (Figure S8). The limit of detection (LoD) was calculated to be 0.15 mM for this sample, with potential for an even better detection limit if lower concentrations are to be tested or greater primary ion doses applied. This is further evidence of the beneficial properties of large doped water clusters in reducing matrix effects.

## Conclusion

This study demonstrates that chemical effects significantly contribute to the effectiveness of large water clusters for SIMS analysis and that primary ion chemistry and velocity (*E/m*) influence both sensitivity and quantification. Using Ar/CO_2_ mixtures as either primary ions or as carrier gases for water clusters, GCIBs predominantly consist of CO_2_ if the input gas is 25% CO_2_ or more. This significantly alters the chemical and physical characteristics of the primary ions compared to pure Ar or pure H_2_O clusters, affecting overall SI yields. We conclude that the differences in CO_2_ percentages used as carrier gases for water GCIBs explain the apparent discrepancies in the literature, as the formation and efficiency of (H_2_O)_n_ clusters (n ∼ 4000) are strongly influenced by the carrier gas composition. Specifically, yields differ significantly between 0% CO_2_ and all other tested ratios (25%, 50%, 75%, and 100% CO_2_). Our data also indicate that the ToF-SIMS performance of large water clusters (n ∼ 26,000) is very sensitive to carrier gas composition. Doping water clusters with 12% CO_2_ increases secondary ion yields for small pharmaceutical compounds, reduces matrix effects, and improves spot size. Each of these results improves the ToF-SIMS imaging capabilities. This supports the principle of customizing ion beams by adjusting carrier gas compositions. Careful consideration and testing are crucial when selecting the most suitable primary ion for a given analysis.

## Supporting Information Available

Materials and methods, experimental beam information, residual gas analyzer data, spot size measurements, doped biological tissue acetaminophen yield comparison, calibration curves, and sodiation/protonation ratios. (PDF)
