# Supplementary material for: Reactive Gas Cluster Ion Beams for Enhanced Drug Analysis by Secondary Ion Mass Spectrometry
Source: Anal Chem. 2024 Sep 13;96(38):15185–93. doi: 10.1021/acs.analchem.4c02144 (PMC11428081; doi:10.1021/acs.analchem.4c02144)
Supplement: Supplementary file 1 — ac4c02144_si_001.pdf [file ac4c02144_si_001.pdf]

Supporting Information

Reactive Gas Cluster Ion Beams for enhanced drug analysis by Secondary Ion Mass Spectrometry

Matija Lagator[1,2], Bilal Patel[1], Sadia Sheraz[1], Nicholas Lockyer[1]*

[1] Department of Chemistry, Photon Science Institute, The University of Manchester, Oxford Road, Manchester, M13 9PL, United Kingdom
[2] Rosalind Franklin Institute, Building R113 Rutherford Appleton Laboratory, Harwell Campus, Didcot, Oxfordshire, OX11 0QX, United Kingdom
[*] Corresponding Author - nick.lockyer@manchester.ac.uk

Cleaning and spin coating details:

The silicon wafer cleaning procedure was different for experiments done with premixed gasses and those done with the Ibeda gas mixer. For the Ibeda gas mixer the cleaning procedure was: solutions of water and methanol were consecutively used twice each, for a duration of 20 minutes. Wafers were allowed to airdry and were then transferred to a UV-Ozone cleaner (Ossila) for 30 minutes. An additional cleaning step was done in the sonication bath with 20 minutes in water followed by 20 minutes in methanol. Once this was completed, the wafers were placed in a new glass vial with pure methanol solution and stored until required. The cleaning procedure for the analysis done with premixed gases: the wafers were placed in a vial and sonicated in chloroform, water, and methanol for an hour each. Once this was completed the wafers were allowed to air dry and were then transferred to a clean glass vial and sealed using parafilm. All three of the solutions (acetaminophen, diclofenac, 1:1 mixture) were spin coated (Laurell Technologies Corporation – Model: WS-400BX-6NPP/LITE) on precleaned silicon wafers that were taken out of the methanol solution and allowed to airdry. A total of 90 µL (5+5+20+30+30 µL) of solution was added using a micropipette The rotational velocity was 500 rpm, and the spin coating was done in static mode (increments of the volume were added to the stationary wafers). Each of the added volume increments was allowed to fully dry prior to addition of more of the solution.

The following is an extended description of how many samples and areas were analysed for each of the following experiments:

1) Analysis of gold using gas mixer - Both $(Ar/CO_2)_n$ and $(H_2O)_n$ – Same thick gold surface for all experiments (but different areas for $(Ar/CO_2)_n$ and $(H_2O)_n$). The surface was first sputtered without analysis in order to remove surface contamination. With gold there was 5 repeated analyses (32×32 pixels) and no need for smaller areas to be selected because vacuum did not affect the gold in the same way it did the drug standards. Total of 5 analysed areas.
2) Drug standards analysed using gas mixer (acetaminophen and diclofenac):
   a. $(Ar/CO_2)_n$– 2 silicon wafer samples - 2 ToF-SIMS analysis regions (32×32 pixels) - 3 areas with (7×7) pixels each were selected for analysis. Total of 12 analysed areas.
   b. $(H_2O)_n$ – 1 silicon wafer sample - 2 ToF-SIMS analysis regions (32×32 pixels) - 2 areas with (7×7) pixels each were selected for analysis. Total of 4 analysed areas.
3) Drug standards analysed using premixed gasses - 1 silicon wafer sample - 6 ToF-SIMS analysis regions (32×32 pixels) - 3 areas with (7×7) pixels each were selected for analysis. Total of 18 analysed areas.
4) Liver tissue sections – 3 ToF-SIMS analysis regions (32×32 pixels) - 3 areas with (7×7) pixels each were selected for analysis. Total of 9 analysed areas.
5) Brain tissue sections – 5 analysis regions per each drug concentration (19×19 pixels) - Total of 5 analysed areas.

Table S1. Experimental parameters for $(Ar/CO_2)_n$ analysis of a gold surface.

Primary ion	$(Ar/CO_2)_n$				
Input Gas Argon Composition (%)	0	25	50	75	100
Energy (keV)	70	70	70	70	70
Average Cluster Size (n)	2139	2150	2127	2098	2114
Energy per nucleon - E/m (eV/nucleon)	0.798	0.794	0.803	0.814	0.807
Current (pA)	95	101	112	70	70
Spot Size (µm)	4.7	4.6	4.6	4.7	4.7
Ion Dose per Layer (ions/cm²)	3.89E+12	4.14E+12	4.59E+12	4.03E+12	4.96E+12
Number of Layers	20	20	20	20	20
Area Analysed (µm²)	300×300	300×300	300×300	300×300	300×300

Table S2. Experimental parameters for $(H_2O)_n$ analysis of a gold surface.

Primary ion	$(H_2O - Ar/CO_2)_n$				
Input Gas Argon Composition (%)	0	25	50	75	100
Energy (keV)	70	70	70	70	70
Average Cluster Size (n)	2526	2342	2275	2401	2577
Energy per nucleon - E/m (eV/nucleon)	1.539	1.661	1.710	1.620	1.509
Current (pA)	283	245	240	233	327
Spot Size (µm)	6.9	7.0	7.1	7.3	7.4
Ion Dose per Layer (ions/cm²)	1.16E+13	1.00E+13	9.83E+12	9.54E+12	1.335E+13
Number of Layers	20	20	20	20	20
Area Analysed (µm²)	300×300	300×300	300×300	300×300	300×300

Table S3. Experimental parameters for $(Ar/CO_2)_n$ analysis of thin drug films (Ace and Diclo).

Primary ion	$(Ar/CO_2)_n$				
Input Gas Argon Composition (%)	0	25	50	75	100
Energy (keV)	70	70	70	70	70
Average Cluster Size (n)	1869	1914	1959	2009	2056
Energy per nucleon - E/m (eV/nucleon)	0.913	0.892	0.872	0.850	0.831
Current (pA)	21	20	21	31	2
Spot Size (µm)	7.6	7.6	8.3	9.4	8.3
Ion Dose per Layer (ions/cm²)	8.12E+10	8.19E+10	8.12E+10	8.47E+10	8.01E+10
Number of Layers	20	20	20	20	20
Area Analysed (µm²)	300×300	300×300	300×300	300×300	300×300

Table S4. Experimental parameters for $(H_2O)_n$ analysis of thin drug films (Ace and Diclo).

Primary ion	$(H_2O - Ar/CO_2)_n$				
Input Gas Argon Composition (%)	0	25	50	75	100
Energy (keV)	70	70	70	70	70
Average Cluster Size (n)	4169	4195	4234	4361	4432
Energy per nucleon - E/m (eV/nucleon)	0.933	0.927	0.918	0.892	0.877
Current (pA)	95	100	101	163	193.5
Spot Size (µm)	5.6	5.5	5.4	5.8	6.2
Ion Dose per Layer (ions/cm²)	2.59E+13	2.76E+13	2.76E+13	4.45E+13	7.91E+13
Number of Layers	20	20	20	20	20
Area Analysed (µm²)	300×300	300×300	300×300	300×300	300×300

Table S5. Experimental parameters for drug film analysis (Ace and Diclo) with a range of PI.

Primary ion	$(H_2O)_n$	$(H_2O)_n$	$(H_2O)_n$
Carrier Gas	Ar/CO_2	Ar	Ar/CO_2
Energy (keV)	70	70	70
Average Cluster Size (n)	9436	25936	25916
Energy per nucleon - E/m (eV/nucleon)	0.412	0.150	0.150
Current (pA)	50	11	18
Spot Size (µm)	13	12	21
Ion Dose per Layer (ions/cm²)	4.06E+11	4.06E+11	4.06E+11
Number of Layers	30	30	30
Area Analysed (µm²)	500×500	500×500	500×500

Table S6. Experimental parameters for analysis of acetaminophen-doped pork liver sections.

Primary ion	C_{60}	$(CO_2)_n$	$(Ar/CO_2)_n$	$(H_2O)_n$	$(H_2O)_n$
Carrier Gas	N/A	N/A	N/A	CO_2	Ar/CO_2
Energy (keV)	40	70	70	70	70
Average Cluster Size (n)	60	11100	11700	23800	25700
Energy per nucleon - E/m (eV/nucleon)	55.556	0.143	0.146	0.163	0.151
Current (pA)	19	2	18	1	30
Spot Size (µm)	9.2	21.0	20.3	18.4	19.9
Ion Dose per Layer (ions/cm²)	4.15E+11	4.10E+11	4.18E+11	3.65E+11	4.1E+11
Number of Layers	30	30	30	30	30
Area Analysed (µm²)	300×300	500×500	300×300	500×500	300×300

Table S7. Experimental parameters for analysis of acetaminophen-doped mouse brain sections.

Primary ion	$(H_2O)n$
Carrier Gas	Ar/CO_2
Energy (keV)	70
Average Cluster Size (n)	26084
Energy per nucleon - E/m (eV/nucleon)	0.149
Current (pA)	20
Spot Size (µm)	14.0
Ion Dose per Layer (ions/cm²)	1.69E+11
Number of Layers	1
Area Analysed (µm²)	540×540

Table S8. Monoisotopic masses and formulas for acetaminophen and diclofenac sodium salt molecular and fragment ions.

Compound	Polarity	Abbreviation	Formula	Monoisotopic mass (g/mol)	Note
Acetamniophen	Positive	[M]	$C_8H_9NO_2$	151.0633	
Acetamniophen	Positive	$[M+H]^+$	$C_8H_{10}NO_2$	152.0712	
Acetamniophen	Positive	$[M+Na]^+$	$C_8H_9NO_2Na$	174.0531	
Acetamniophen	Positive	$[M-H+2Na]^+$	$C_8H_8NO_2Na_2$	196.0350	
Acetamniophen	Positive	$[2M+H]^+$	$C_{16}H_{19}N_2O_4$	303.1345	
Acetamniophen	Positive	$[2M+Na]^+$	$C_{16}H_{18}N_2O_4Na$	325.1164	
Acetamniophen	Positive	$[2M+H]^+$	$C_{16}H_{19}N_2O_4$	303.1345	
Acetamniophen	Positive	$[2M+Na]^+$	$C_{16}H_{18}N_2O_4Na$	325.1164	
Acetamniophen	Positive	$[F]^+$	C_6H_7NO	109.0528	
Acetamniophen	Positive	$[F+H]^+$	C_6H_8NO	110.0606	
Acetamniophen	Negative	$[M-H]^-$	$C_8H_8NO_2$	150.0555	
Acetamniophen	Negative	$[2M-H]^-$	$C_{16}H_{17}N_2O_4$	301.1188	
Acetamniophen	Negative	$[F-H]^-$	C_6H_5NO	107.0371	
Diclofenac Sodium Salt	Positive	[M]	$C_{14}H_{10}NO_2Cl_2Na$	316.9986	
Diclofenac Sodium Salt	Positive	$[M+H]^+$	$C_{14}H_{11}NO_2Cl_2Na$	318.0065	Isotopic peaks at 320, 322, 324
Diclofenac Sodium Salt	Positive	$[M+Na]^+$	$C_{14}H_{10}NO_2Cl_2Na_2$	339.9884	Isotopic peak at 342
Diclofenac Sodium Salt	Positive	$[M-Na+2H]^+$	$C_{14}H_{12}NO_2Cl_2$	296.0245	Isotopic peak at 298
Diclofenac Sodium Salt	Positive	$[F]^+$	$C_{13}H_9NCl$	214.0424	Isotopic peak at 216
Diclofenac Sodium Salt	Negative	$[M-H]^-$	$C_{14}H_9NO_2Cl_2Na$	315.9908	Isotopic peak at 318
Diclofenac Sodium Salt	Negative	$[M-Na]^-$	$C_{14}H_{10}NO_2Cl_2$	294.0089	Isotopic peak at 296
Diclofenac Sodium Salt	Negative	$[M-CO_2-Na]^-$	$C_{14}H_{10}NO_2Cl_2$	250.0190	Isotopic peak at 252
Diclofenac Sodium Salt	Negative	$[F]^-$	$C_{13}H_9NCl$	214.0424	Isotopic peak at 216

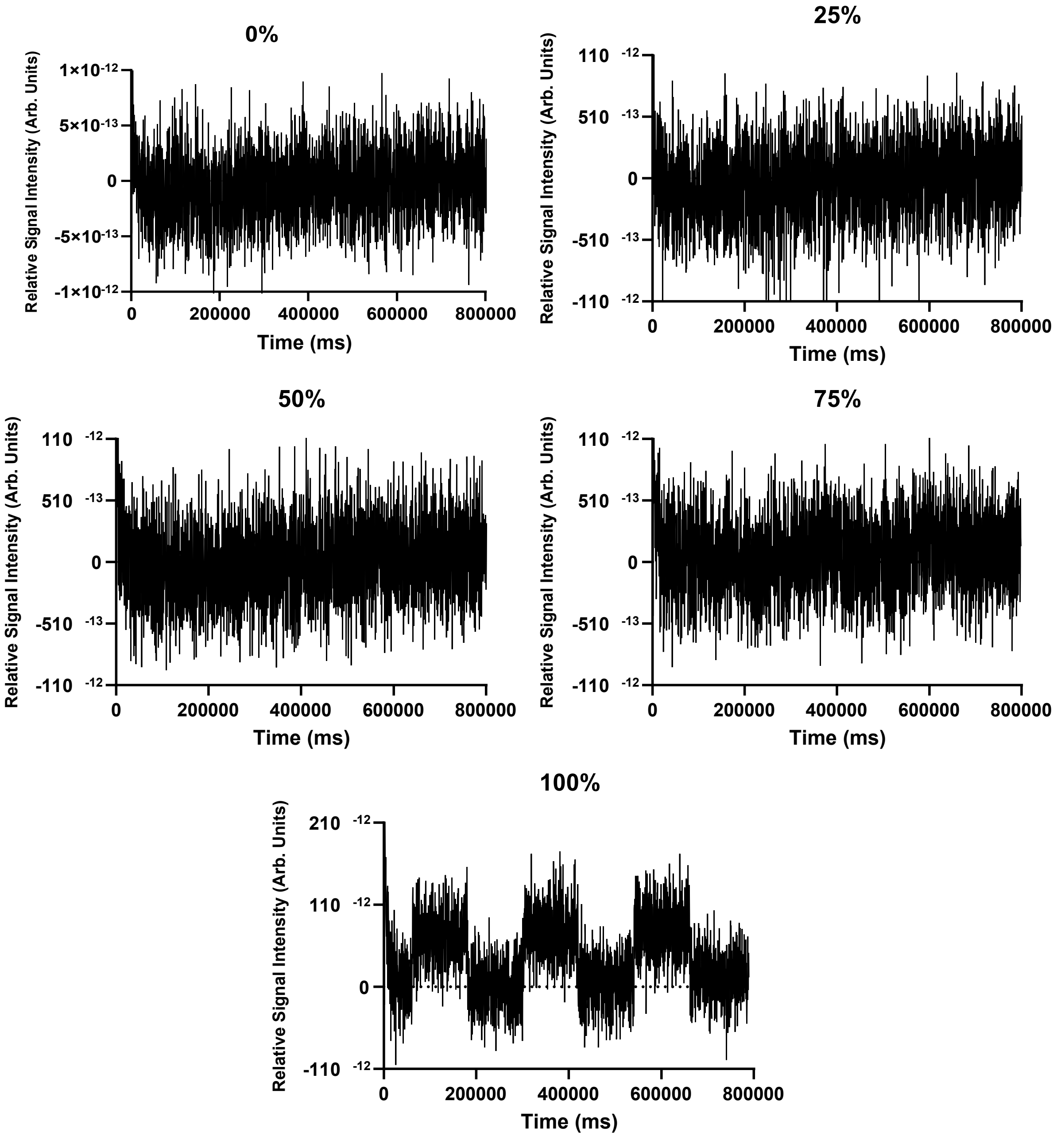

Figure S1. Showing the raw *m/z* 40 data for the RGA analysis of $(ArCO_2)_n$ clusters. The percentages represent the amount of argon in the input gas. From the 100% dataset clear differences can be observed in signal intensity when the gate valve is opened (higher signal) or closed (lower signal). This difference is not as clear in the other datasets. The averages of open and closed signal intensities are used to plot Figure S2.

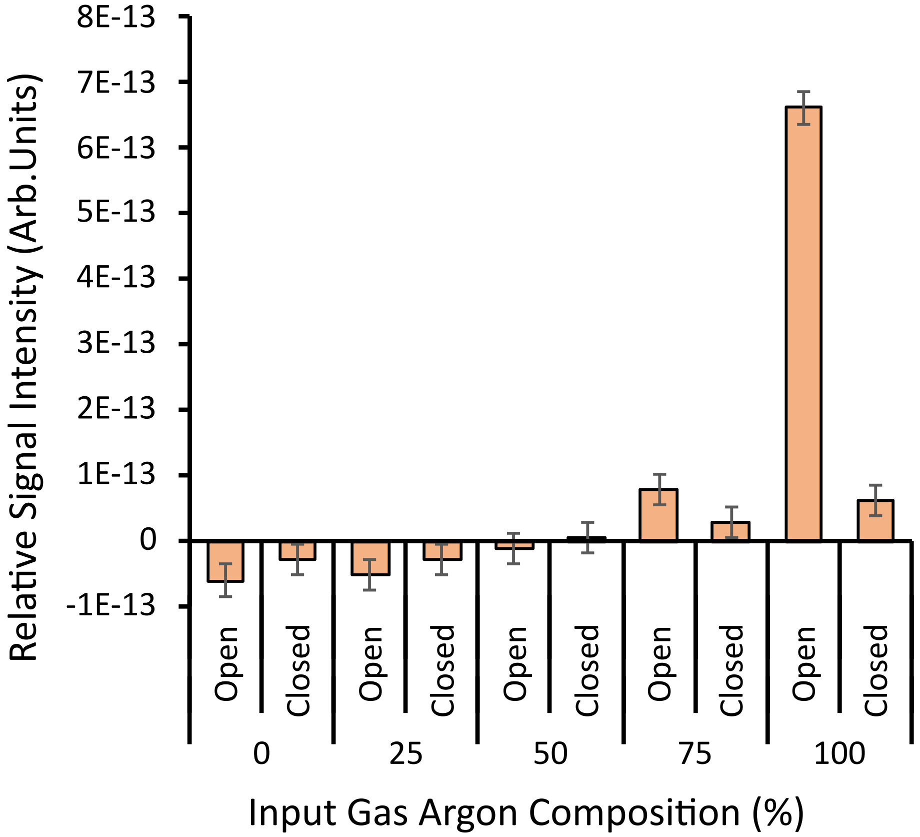

Figure S2. Residual gas analyser results for $(Ar/CO_2)_n$ clusters with varying ratios of Ar and CO_2. Signal intensity shown is for *m/z* 40. Data shown here is the average of open and closed time periods shown in Figure S1.

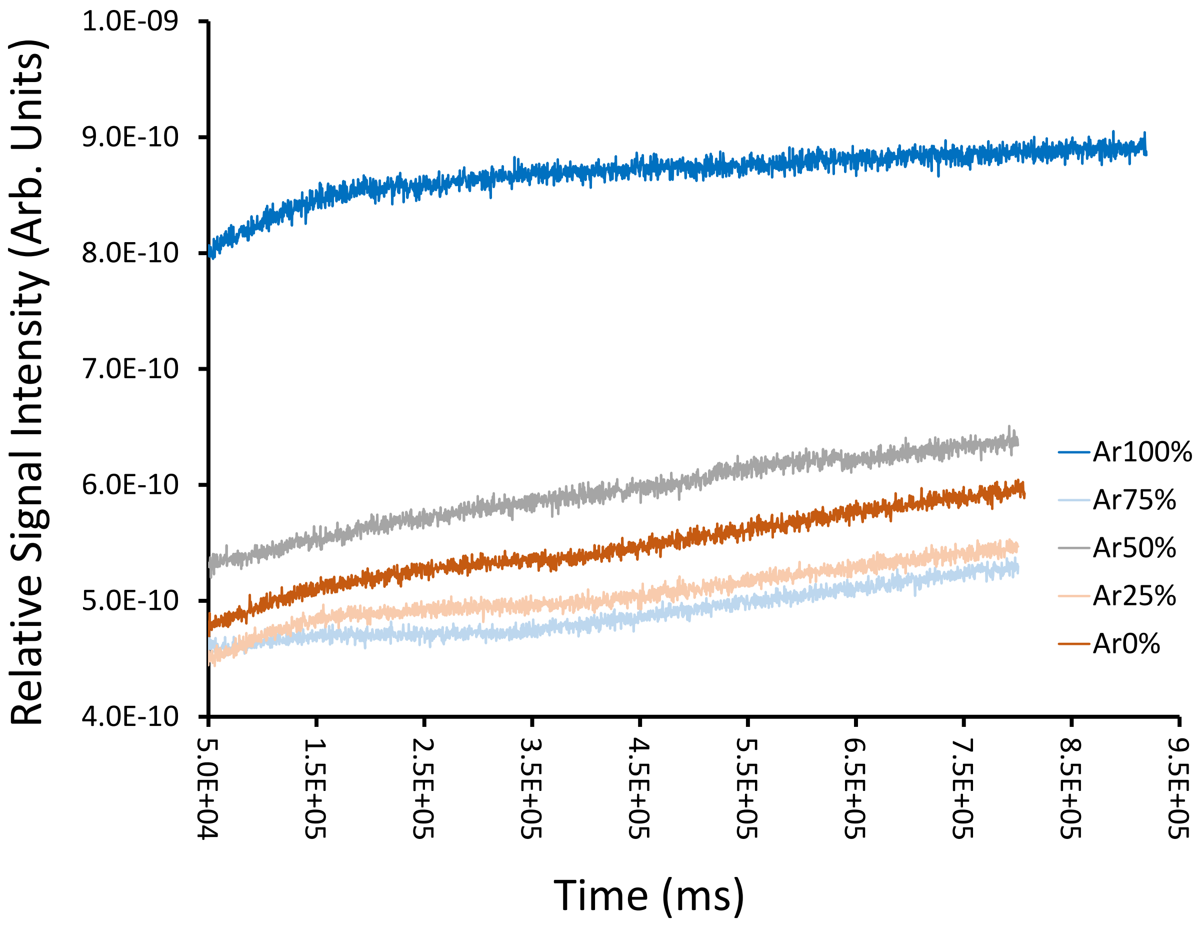

Figure S3. Showing the raw *m/z* 18 data for the RGA analysis of $(H_2O)_n$ clusters. The percentages represent the amount of argon in the carrier gas. No clear differences can be observed in signal intensity when the gate valve is opened or closed.

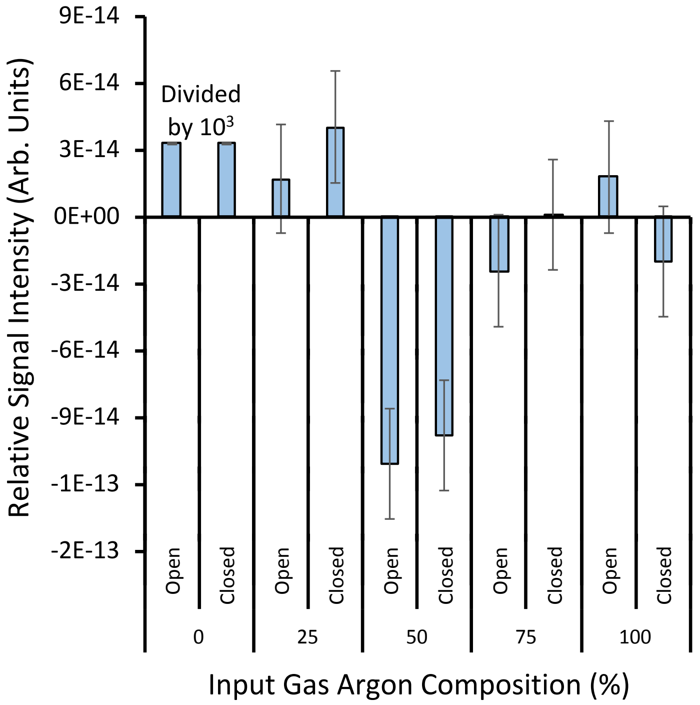

Figure S4. Residual gas analyser results for water clusters with varying ratios of Ar and CO_2 as the carrier gas. Signal intensity shown is for *m/z* 40, except for 0% input argon which shows *m/z* 44 signal intensity. This is also the explanation why this dataset had to be divided by 10^3.

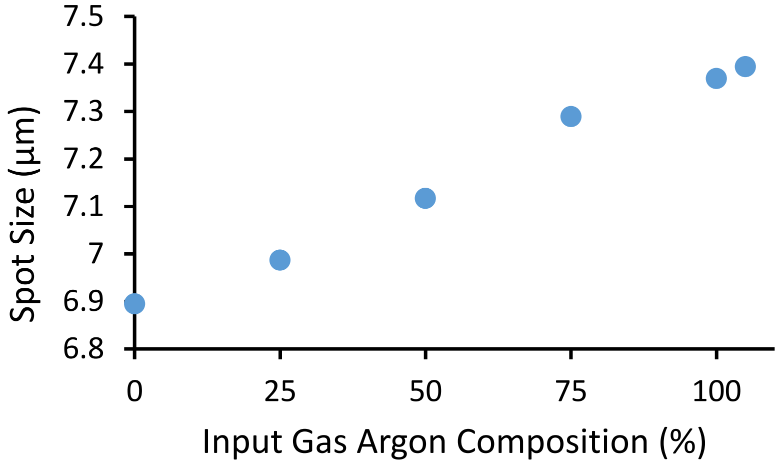

Figure S5. Showing the correlation between spot size and carrier gas composition for water clusters. These results were obtained using the varioMix gas mixer. Note that the two values at 100% are both obtained using 100 % Ar carrier gas but were done at the start and end of analysis as a control.

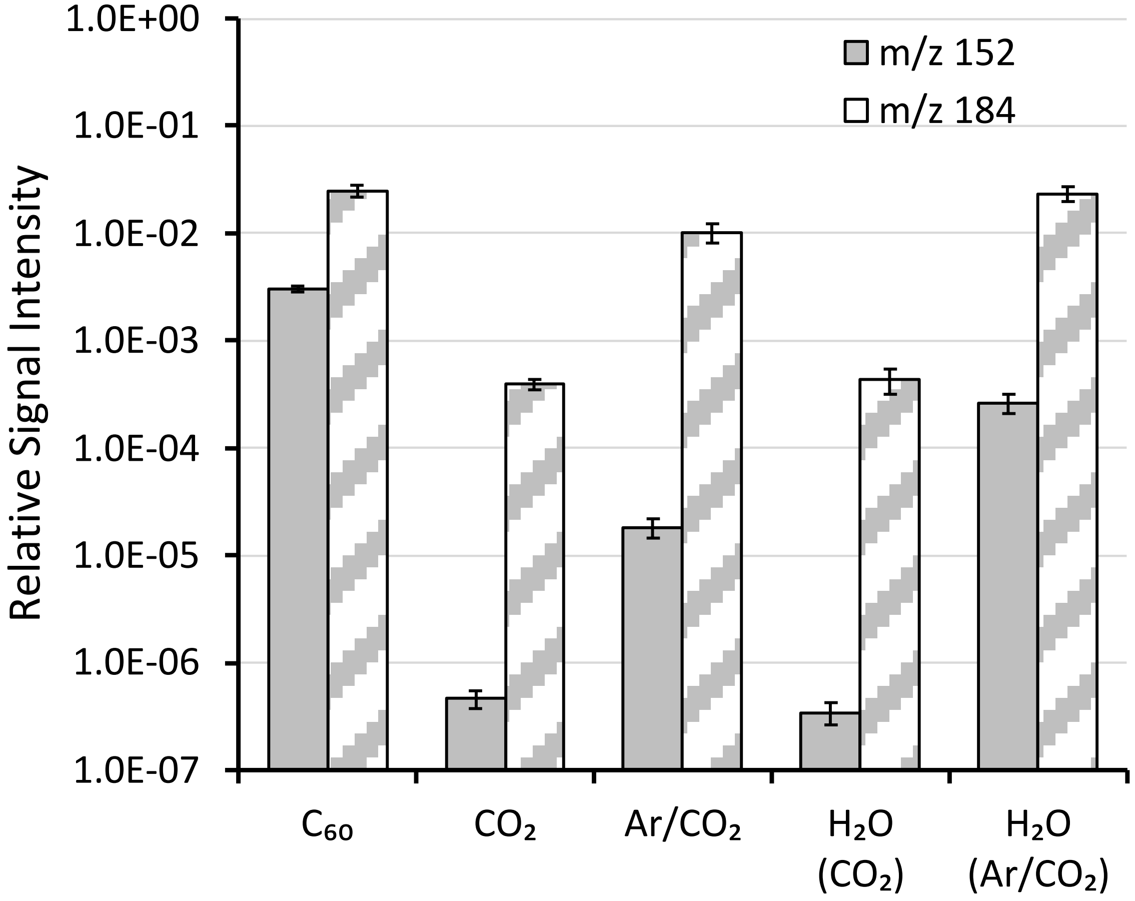

Figure S6. Comparing acetaminophen (*m/z* 152) and phosphatidylcholine (PC) headgroup (m/z 184) yields, obtained from drug-doped pork liver tissue. Five different primary ions are compared. The experimental parameters are shown in Table S6. The *E/m* value for all of the cluster ions was ~0.15. These values are the sum of peaks for all 6 drug concentrations used to generate calibration curves (0, 0.625, 1.25, 2.5, 5, 10 mM).

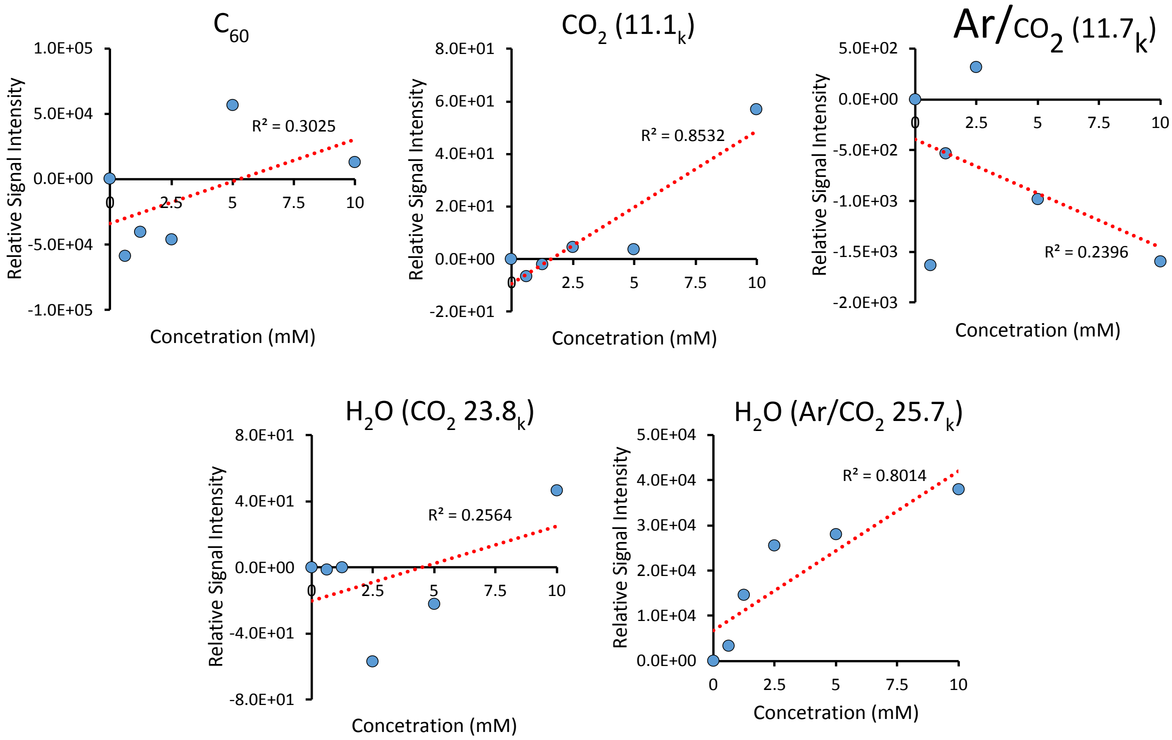

Figure S7. Acetaminophen calibration curves (*m/z* 152) for 5 different primary ions. Acetaminophen (3 µL) in different concentrations was doped onto pork liver sections (each section was doped with a different concentration of drug). Comparing the efficiency of the PIs to detect pharmaceuticals in a quantitative manner. Values for each PI were control subtracted in order to correct for the background levels of signal at *m/z* 152.

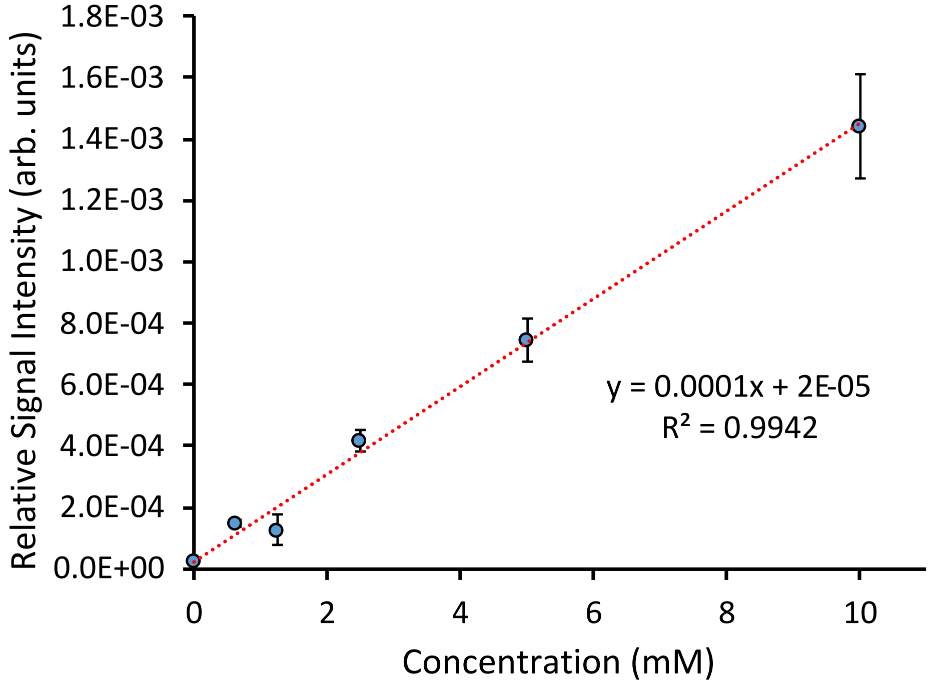

Figure S8. Calibration curve for acetaminophen protonated molecular ion peak (*m/z* 152.07) on mouse brain tissue. Drug was doped onto the tissue and analysed using ToF-SIMS water clusters - $(H_2O)_{26000}$. Five different areas on the doped tissue were selected for analysis. Limit of detection (LoD) was calculated to be approximately 0.15 mM.

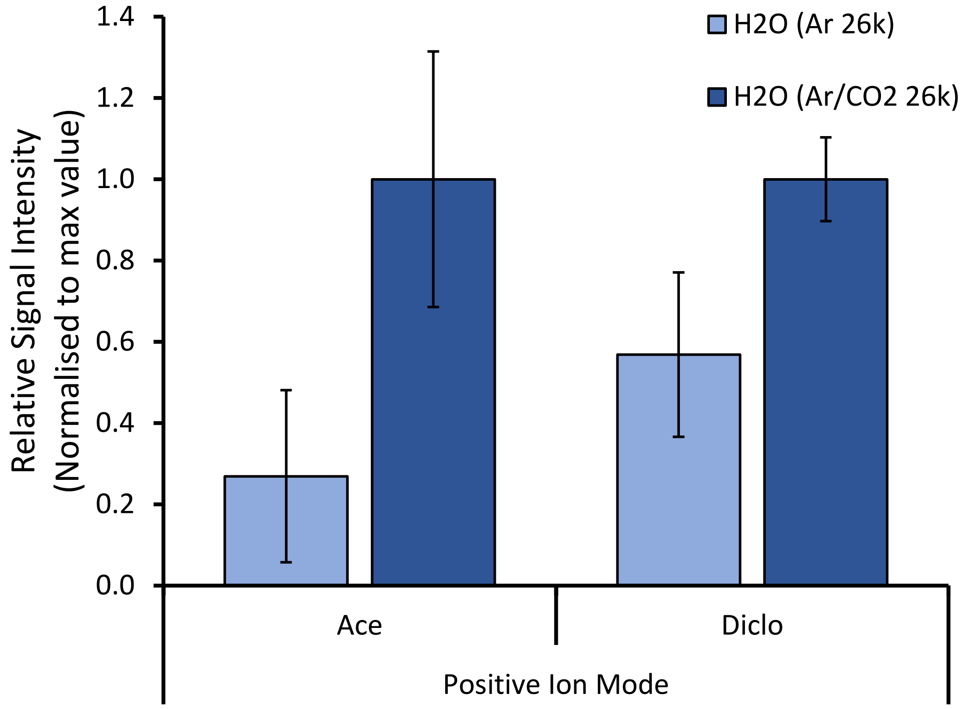

Figure S9. Comparing the ratio of sodiated to protonated peaks for acetaminophen and diclofenac sodium salt. Samples were analysed using large water GCIB (approximately 26000 water molecules), with either pure Ar or Ar/CO_2 carrier gas.
